# Treatment with the apoptosis inhibitor Asunercept reduces clone sizes in patients with lower risk Myelodysplastic Neoplasms

**DOI:** 10.1007/s00277-024-05664-5

**Published:** 2024-02-27

**Authors:** Alexander Streuer, Johann-Christoph Jann, Tobias Boch, Maximilian Mossner, Vladimir Riabov, Nanni Schmitt, Eva Altrock, Qingyu Xu, Marie Demmerle, Verena Nowak, Julia Oblaender, Iris Palme, Nadine Weimer, Felicitas Rapp, Georgia Metzgeroth, Anna Hecht, Thomas Höger, Christian Merz, Wolf-Karsten Hofmann, Florian Nolte, Daniel Nowak

**Affiliations:** 1 Department of Hematology and Oncology, Medical Faculty Mannheim of the Heidelberg University, Pettenkoferstr. 22, 68169 Mannheim, Germany; 2 Centre for Genomics and Computational Biology, Barts Cancer Institute, London, UK; 3 Apogenix GmbH, Heidelberg, Germany

**Keywords:** Myelodysplastic Neoplasms, Asunercept, APG101, Targeted therapies, Apoptosis inhibitor

## Abstract

In low-risk Myelodysplastic Neoplasms (MDS), increased activity of apoptosis-promoting factors such as tumor necrosis factor (TNFα) and pro-apoptotic Fas ligand (CD95L) have been described as possible pathomechanisms leading to impaired erythropoiesis. Asunercept (APG101) is a novel therapeutic fusion protein blocking CD95, which has previously shown partial efficacy in reducing transfusion requirement in a clinical phase I trial for low-risk MDS patients (NCT01736436; 2012-11-26). In the current study we aimed to evaluate the effect of Asunercept therapy on the clonal bone marrow composition to identify potential biomarkers to predict response. Bone marrow samples of *n* = 12 low-risk MDS patients from the above referenced clinical trial were analyzed by serial deep whole exome sequencing in a total of *n* = 58 time points. We could distinguish a mean of 3.5 molecularly defined subclones per patient (range 2–6). We observed a molecular response defined as reductions of dominant clone sizes by a variant allele frequency (VAF) decrease of at least 10% (mean 20%, range: 10.5–39.2%) in dependency of Asunercept treatment in 9 of 12 (75%) patients. Most of this decline in clonal populations was observed after completion of 12 weeks treatment. Particularly early and pronounced reductions of clone sizes were found in subclones driven by mutations in genes involved in regulation of methylation (*n* = 1 *DNMT3A*, *n* = 1 *IDH2*, *n* = 1 *TET2*). Our results suggest that APG101 could be efficacious in reducing clone sizes of mutated hematopoietic cells in the bone marrow of Myelodysplastic Neoplasms, which warrants further investigation.

**Supplementary Information:**

The online version contains supplementary material available at 10.1007/s00277-024-05664-5.

## Introduction

Myelodysplastic Neoplasms (MDS) are diseases of the hematopoietic stem cell stem- and progenitor cells (HSPCs) and are characterized by dysplasia in the bone marrow and blood resulting in hematopoietic insufficiency.

In this context, anemia represents one of the most common clinical symptoms, which can currently either be treated symptomatically by red blood cell concentrates (RBC) or by enhancing erythroid output of the bone marrow, e.g. by erythroid stimulating agents (ESA) or targeted agents aimed at specific molecular pathomechanisms in MDS [1]. To this end, the newly approved TGFβ ligand trap Luspatercept has recently emerged as a therapeutic option able to induce complete or partial remissions, albeit frequently with limited duration [2]. Therefore, there is still an unmet need for further targeted treatment options for anemia in lower risk MDS.

The production of red blood cells is promoted by the hormone erythropoietin (EPO) in response to tissue hypoxia [3]. As part of a negative regulation of this process, immature erythroid cells express several death receptors, whose ligands are produced by mature erythroblasts. The accumulation of mature erythroblasts may moderate the development of immature erythroid cells through death-receptor triggering and caspase activation resulting in differentiation blockade and apoptosis [4]. As a possible molecular pathomechanism for anemia in low-risk MDS, a pro-apoptotic niche with increased activity of apoptosis-promoting factors such as tumor necrosis factor (TNFα) and pro-apoptotic Fas ligand (CD95L) have been proposed [4–8]. CD95 (Fas receptor, also known as Fas, FasR, APO-1, APT1 or TNFRSF6) is a member of the TNF (tumor necrosis factor) death receptor superfamily. By binding of its ligand CD95L (Fas ligand, FasL or CD178), CD95 activation leads to recruitment of intracellular death domain containing adaptors such as FAS-associated death domain (FADD) and TNFR-associated death domain (TRADD), which initiate the caspase cascade and subsequently induce apoptosis [9].

There is evidence that the CD95 pathway is essential to regulate erythrocyte, neutrophil and megakaryocyte differentiation by inducing cell death in immature progenitor cells by eliminating auto-reactive B lymphocytes and controlling T cell homeostasis [4, 10]. In accordance with this, a recent study showed that the inhibition of CD95 signaling rescued burst-forming unit-erythroid (BFU-E) growth from early stage MDS-derived CD34+ progenitors without impairing erythroid differentiation [11].

Asunercept (APG101) is a novel therapeutic fusion protein, consisting of the extracellular domain of human CD95 and the Fc region of human IgG1, blocking the interaction between CD95 and its ligand. In in-vitro studies of primary MDS samples Asunercept was able to increase the number of (BFU-E) progenitors derived from CD34+ progenitors in liquid culture and rescued BFU-E growth by inhibiting apoptosis. These effects were independent of CD95 or CD95L expression levels [12]. Moreover, CD95 overexpression was associated with an ESA resistance [12].

In a recently published clinical phase I trial (NCT01736436), Asunercept treatment was assessed in low and intermediate-risk MDS patients [13]. Transfusion requirement as a secondary endpoint was reduced without an increase of the frequency or severity of adverse events. The need for transfusion of responding patients (*n* = 9 of 20 patients, 45%) was decreased from a mean of 11.4 [± 3.9] to 9.33 [± 3.5] packed red blood cells (pRBCs) within twelve weeks, and to 8.56 [± 3.5] within the next consecutive 12-weeks.

Based on these clinical findings, we now aimed to further investigate the underlying mechanisms of action of APG101 and to uncover the molecular differences between responders and non-responders. Therefore, we assessed the effect of Asunercept therapy on the clonal composition in MDS patients enrolled in this trial [13] in order to identify susceptible clones and subclones and to determine molecular biomarkers. Deep sequencing of a particularly large number of time points in the treatment course of MDS patients with Asunercept was investigated and clonal evolution was assessed by serial whole exome sequencing (WES) as previously described [14]. In addition, molecular changes were correlated with clinical parameters such as hemoglobin levels and transfusion burden.

## Methods

### Patients

For the current molecular study, *n* = 12 MDS patients from the clinical phase I study as performed by Boch et al. (NCT01736436) [13] were included. Detailed information about the design of the clinical trial can be found in the publication [13] and in the patient cohort **(**Table 1**)** and patient characteristics table **(**Table 2**)**. In brief, it was a prospective, open-label, single-arm phase I study which tested the safety, tolerability and pharmacodynamic effects of Asunercept on erythropoiesis in transfusion-dependent MDS patients.

**Table 1:** Patient cohort

Pat_ID	Sex	Age	Dose group	WHO class. (2016)	IPSS-R (score)	Cytogenetics	Blast %BM	Hb [g/dl]	Plt [n/nl]	WBC [n/nl]	ECOG
Clinical responders											
P01-01	m	78	400 mg	MDS-RS-MLD	Very low (1)	46,XY,del(12)(p11p13) [5]; 46,XY [15]	1–2%	11	217	3.61	1
P01-05	m	76	100 mg	MDS-EB-1	Intermediate (4.5)	46,XY [20]	5%	8.4	86	2.22	1
P01-06	f	64	100 mg	MDS-del(5q)	Low (3)	46,XX,del(5)(q14q34), del(20)(q11q13) [14] 46,XX [6]	<5%	9.3	42	3.1	0
P01-10	m	82	100 mg	MDS-MLD	Very low (1.5)	45,X,-Y [20]	<1%	7.1	113	3.26	0
P01-12	m	62	100 mg	MDS-MLD	Low (2)	46,XY [20]	<2%	10.5	32	3.13	1
Clinical non-responders											
P01-02	f	76	400 mg	MDS-MLD	Low (2)	46,XX [15]	<1%	11.5	21	7.39	2
P01-11	m	76	100 mg	MDS-MLD	Intermediate (4)	46,XY [20]	2–3%	7	59	1.8	0
P01-15	f	74	100 mg	MDS-MLD	Intermediate (4.5)	46,XX,del(9)(q21q34) [13]; 46,XX [7]	<5%	9.3	100	1.67	0
P01-16	f	56	100 mg	MDS-RS-MLD	Intermediate (4)	46,XX [20]	<5%	9.2	260	5.71	0
P01-17	m	74	100 mg	MDS-MLD	Low (2)	46,XY [20]	1–2%	10	82	1.17	1
P01-18	m	75	100 mg	MDS-MLD	Very low (1.5)	46,XY [20]	<1%	10	63	3.31	1
P01-20	m	76	100 mg	MDS-MLD	Intermediate (4.5)	46,XY [20]	<5%	9	28	1.62	2

All patients were recruited and treated as described in [13]. *N* = 12 patients with a long follow-up were selected for this study. There was no significant difference between the groups

*WHO* World Health Organization, *IPSS-R* revised international prognostic scoring system, *blast%BM* percentage of blasts in bone marrow, *Hb* hemoglobin, *Plt* platelets count, *WBC* white blood cells count, *MDS-RS-MLD* MDS with multiple lineage dysplasia and ring sideroblasts, *MDS-MLD* MDS with multilineage dysplasia, *MDS-EB-1* MDS with excess blasts 1, *MDS-del(5q)* MDS with a 5q-deletion

**Table 2:** Patient characteristics

	All *n* = 12	Clinical responder *n* = 5	Clinical non-responder *n* = 7	*p*-value
Age				
Median (range)	72.4 (56–82)	72.4 (62–82)	72.4 (56–76)	0.99
Sex, *n* (%)				
Male	8 (66.7%)	4 (80%)	3 (42.9%)	0.58
Female	4 (33.3%)	1 (20%)	4 (57.1%)	
ECOG *n* (%)				
0	5 (41.7%)	2 (40%)	3 (42.9%)	0.75
1	5 (41.7%)	3 (60%)	2 (28.6%)	
2	2 (16.7%)	0 (0%)	2 (28.6%)	
3				
WHO class. (2016), *n* (%)				
MDS-MLD	8 (66.7%)	2 (40%)	6 (85.7%)	0.36
MDS-RS-MLD	2 (16.7%)	1 (20%)	1 (14.3%)	
MDS del(5q)	1 (16.7%)	1 (20%)	0	
MDS-EB-1	1 (8.3%)	1 (20%)	0	
IPSS-R risk (*n*)				
Very low risk	3 (25.0%)	2 (40%)	1 (14.3%)	0.47
Low	4 (33.3%)	2 (40%)	2 (28.6%)	
Interm. risk	5 (41.7%)	1 (20%)	4 (57.1%)	
Hb [g/dL]				
Median (range)	9.4 (7–11.5)	9.3 (7.1–11)	9.4 (7–11.5)	0.85
WBC [×10^9]				
Median (range)	3.2 (1.17–7.39)	3.1 (2.22–3.61)	3.2 (1.17–7.39)	0.86
Plt [×10^9]				
Median (range)	91.9 (21–260)	98 (32–217)	87.6 (21–260)	0.82
BM blast [%]				
<2%	5 (41.7%)	3 (60%)	2 (28.6%)	0.09
=<2%–<5%	5 (41.7%)	0 (0%)	5 (71.4%)	
=<5%–10%	1 (8.3%)	1 (20%)	0 (0%)	
na	1 (8.3%)	1 (20%)	0 (0%)	
EPO [U/L]				
Median (range)	708 (30–3499)	579 (30–1736)	800 (59–3499)	0.76

Comparison of the clinical parameters of the responder group versus the non-responder group. The Wilcoxon signed-rank test was used as a significance test for continuous variables. For categorical variables, the Fisher’s exact test was used

*ECOG* Eastern Cooperative Oncology Group, *EPO* erythropoietin

### Whole exome sequencing of serial time points

**Fig. 1:**
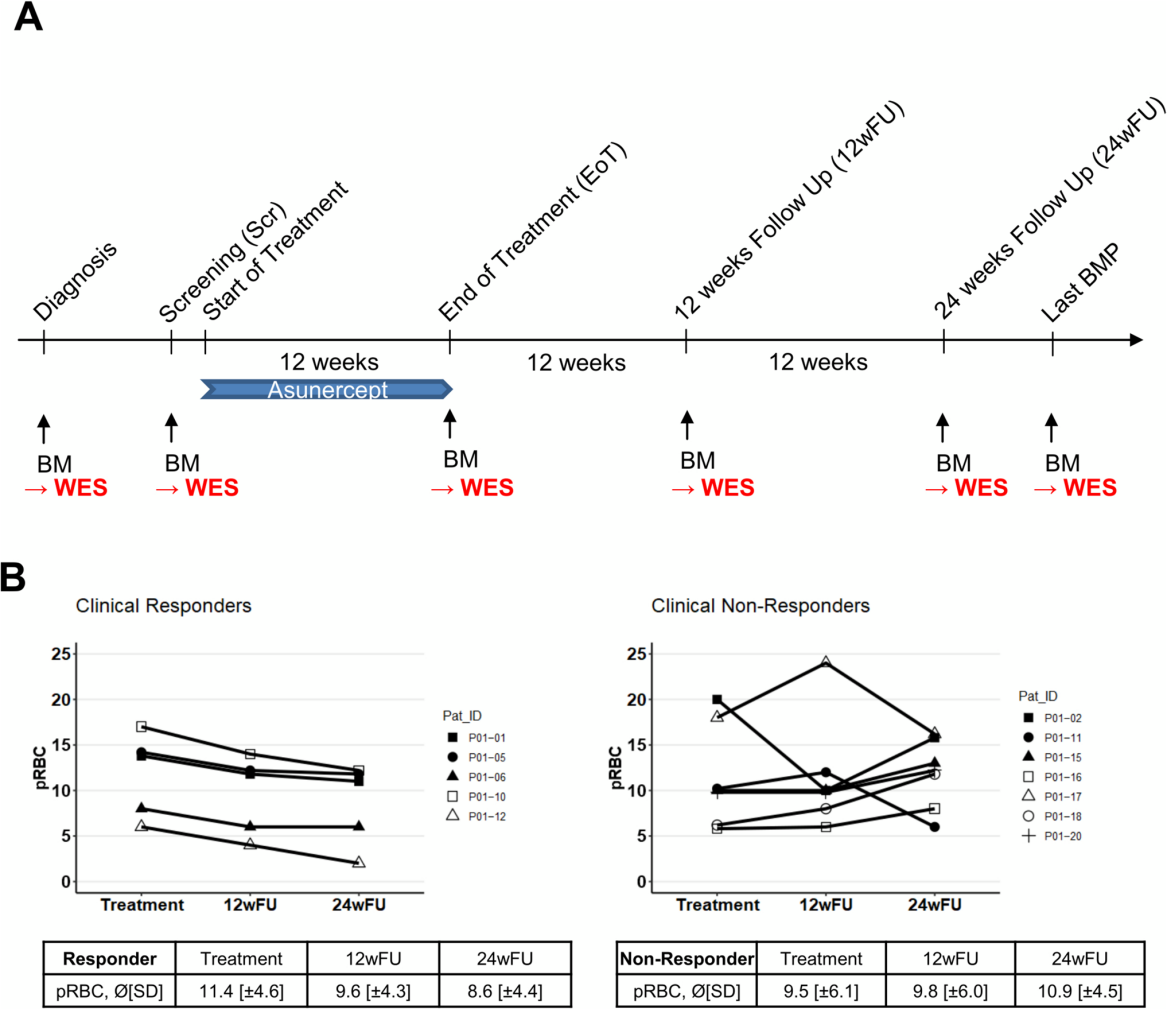
Study design and results of preceding study. **a** Asunercept (APG101) was administered intravenously once a week at a dose of 400 mg (*n* = 2) or 100 mg (*n* = 12). Following an initial screening, treatment was conducted for 12 weeks. The follow-up period lasted further 24 weeks. Bone marrow (BM) aspirates were performed for screening (Scr), end of treatment (EoT), after 12 (12wFU) and after 24 weeks (24wFU) of follow-up according to [13]. Additional material from initial diagnosis and last follow-up were used and subjected to Whole Exome Sequencing (WES). **b** A continuous decrease in transfusion frequency was defined as response. In *n* = 5 patients of the current study, a decline from 11.4 Units of packed red blood cells (pRBC) transfusions during the 12-week treatment to 9.6 transfusions during week 13 to week 24 (12-week follow-up, 12wFU) and 8.6 pRBC transfusions during week 25 to week 36 (24-week follow-up, 24wFU) was observed (*p* = 0.058). These patients are in the following referred as responder. In *n* = 9 patients there was no change in transfusion frequency, these are referred as non-responder

Serial bone marrow biopsies (BMP) were performed within the clinical trial NCT01736436 for screening (Scr), end of treatment (EoT), 12 weeks after EoT (12wFU) and 24 weeks after EoT (24wFU). Additional biopsies for the current study were obtained from initial diagnosis and further follow-up samples if available **(**Fig. 1a**)**. In total, *n* = 58 chronological bone mononuclear marrow cells samples (MNCs) from 12 patients were subjected to whole exome sequencing: Thereby *n* = 1 patient had three available time points, *n* = 5 patients had four available time points, *n* = 2 patients had five available time points, *n* = 3 patients had 6 available time points and *n* = 1 patient had seven available time points. DNA from matched bone marrow derived stroma cells (MSCs) served as germline controls.

For library preparation the Illumina DNA Flex for Enrichment Kit with probes from the IDT xGen Exome Research panel was used with 150 ng DNA input.

Sequencing was performed on an Illumina NovaSeq 6000 platform in the 150 bp paired-end configuration. The mean coverage was 92.7× with a range of 44.4× to 164× per sample.

### Bioinformatical analysis

The sequencing data was mapped with *bwa* v0.7.5 and then deduplicated and recalibrated using *Picard* v1.100 and the *GATK* toolkit v3.8 [15–17]. For quality control, the tools *Fastqc* v0.11.9 and *Qualimap* v2.2.1 were used [18]. Somatic point-mutations and small Indel mutations were queried with *Mutect2* v4.1.3.0 [19]. Variants with a coverage less than 15 reads, a VAF less than 5% in tumor sample and a VAF in the germline control above 1% were excluded from analysis. Copy number changes and large structural chromosomal aberrations were investigated with the *Sequenza* v2.1.0 [20] tool. Clustering of mutations and reconstruction of individual clones was performed with *SciClone* v1.1.0 [21]. Graphical images were generated with the R package *ggplot2* [22]. Mutational hierarchies were plotted as “Fishplots”, which were generated with the R package *fishplots* [23].

### Statistical analysis

Continuous variables were summarized by means of descriptive statistics in summary tables, i.e., by the number of observations, arithmetic mean, standard deviation, minimum, median and maximum. Categorical data were summarized by means of frequency tables, i.e. counts and percentages. Concerning the descriptive nature of the analyses, the comparison of the transfusion burden was performed by using a matched-pairs Wilcoxon signed rank test as a post-hoc analysis. In general, the Wilcoxon signed-rank test was used as a significance test for continuous variables. For categorical variables, the Fisher’s exact test was used. Statistical significance was assumed at *p* ≤ 0.05.

## Results

### Assessment of transfusion requirement following 12 weeks of treatment with Asunercept and definition of clonal molecular response

In the previous phase I clinical trial assessing safety and tolerability of Asunercept in low risk MDS (NCT01736436) [13] none of the patients reached erythroid response as defined by IWG criteria. Nevertheless, the aim of this current study was to gain insights into possible effects on the molecular level in the bone marrow of MDS patients treated with Asunercept. To this end, patients were separable into a group with continuous decrease of transfusion burden between the 1st and 12th week (12wFU) and the 13th and 24th week (24wFU) of follow-up and classified as “clinical responders” versus a group of patients, who displayed stable or increased transfusion requirement of pRBCs classified as “clinical non-responders”. For this study, *n* = 5 patients were categorized as “clinical responders” and *n* = 7 patients as “clinical non-responders” **(**Fig. 1b**)**.

The focus of this study was to identify molecular changes in the bone marrow. A reduction of the dominant clone by a relative proportion of more than 10% between EoT and 12wFU was defined as a molecular responder. Patients not meeting this criterion were classified as molecular non-responders. A VAF (Variant Allele Frequency) reduction of over 10% was chosen to ensure the exclusion of technical fluctuations. Moreover, a threshold of 10% VAF is the minimum requirement for molecular classification within the new 2022 WHO and ICC classifications [24] and has been used as a marker of molecular response in previous clinical trials, e.g. driver mutation reduction in myelofibrosis [25].

### Molecular characterization of clinical responders and non-responders to Asunercept therapy

To investigate whether Asunercept treatment induces changes of the clonal composition, we performed whole exome sequencing (WES) of sequential bone marrow samples from MDS patients and non-hematopoietic MSCs as a germ line controls to identify potential biomarkers.

**Fig. 2:**
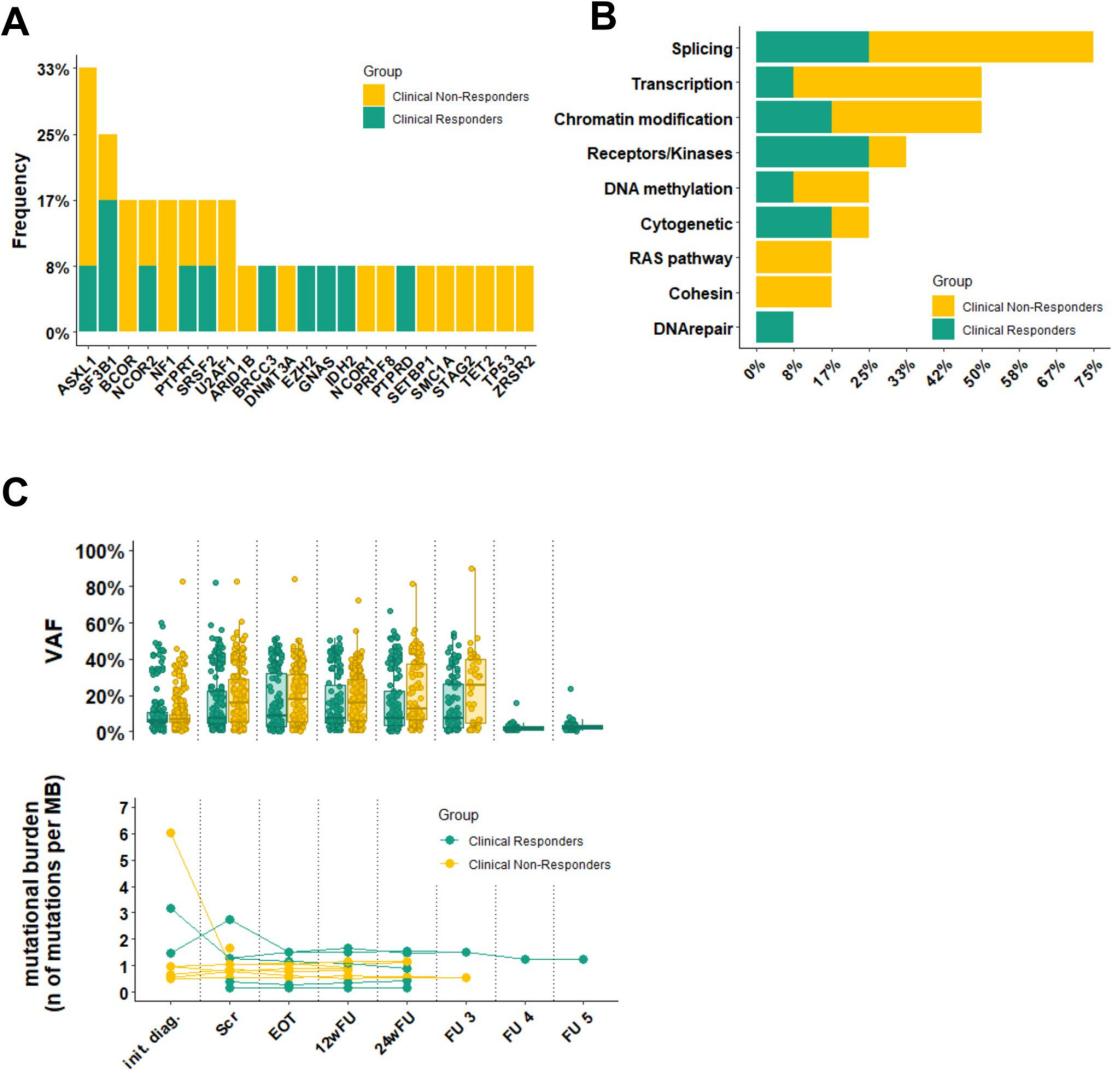
Molecular characterization between responders and non-responders to treatment with Asunercept. **a** Frequency of recurrently mutated genes in myeloid neoplasia (*n* = 23) separated by responder vs non-responder; *ASXL1* was the most frequently mutated gene (*n* = 4), followed by *SF3B1* (*n* = 3). *P*-values for the first 8 genes: ASXL1: 0.58, SF3B1: 0.522, BCOR, NF1, U2AF1: 0.47, NCOR2, PTPRT, SRSF2: 1.0 **b** The most affected cellular process was the spliceosome machinery (*n* = 9) in our cohort. *P*-values: Splicing: 0.52, Transcription: 0.24, Chromatin modification: 1.0, Receptors/Kinases: 0.22, DNA methylation: 1.0, Cytogenetic: 0.52, RAS pathway, Cohesin: 0.47, DNA repair: 0.42 C: The VAF (variant allele frequency) and number of mutations per MB (megabase) in the course of time; on average, a patient carried 0.95 mutations per megabase (range: 0.1 to 2.7) at the screening timepoint. init. diag.: initial diagnosis; Scr: screening; EOT: End of Treatment; 12wFU: 12 weeks follow-up; 24wFU: 24 weeks follow-up; FU 3: third follow-up (week 91 and 171); FU 4; fourth follow-up (week 230); FU 5 (week 297): fifth follow-up

In total we detected somatically acquired mutations in 787 genes in our cohort. Of these, 23 genes are known as recurrently mutated in myeloid neoplasia [26]. *ASXL1* was the most frequently mutated gene in *n* = 4 cases, followed by *SF3B1* in *n* = 3 cases **(**Fig. 2a**)**. Mutated genes were grouped by Gene ontology (GO) pathways [26] or primary literature sources. The most affected cellular process by mutations was the spliceosome machinery with *n* = 9 (64%) cases in splice factor genes such as *SF3B1, U2AF1, SRSF2* and *ZRSR2* **(**Fig. 2b**)**.

On average, the mutational burden per MDS patients in this study was 0.95 mutations per megabase (range: 0.1 to 2.7) at the screening timepoint. *N* = 2 patients had more mutations in terms of quantity at the time of initial diagnosis with no previous treatment with concomitant lower variant allele frequencies (VAF) than later in the time course (Fig. 2c).

**Fig. 3:**
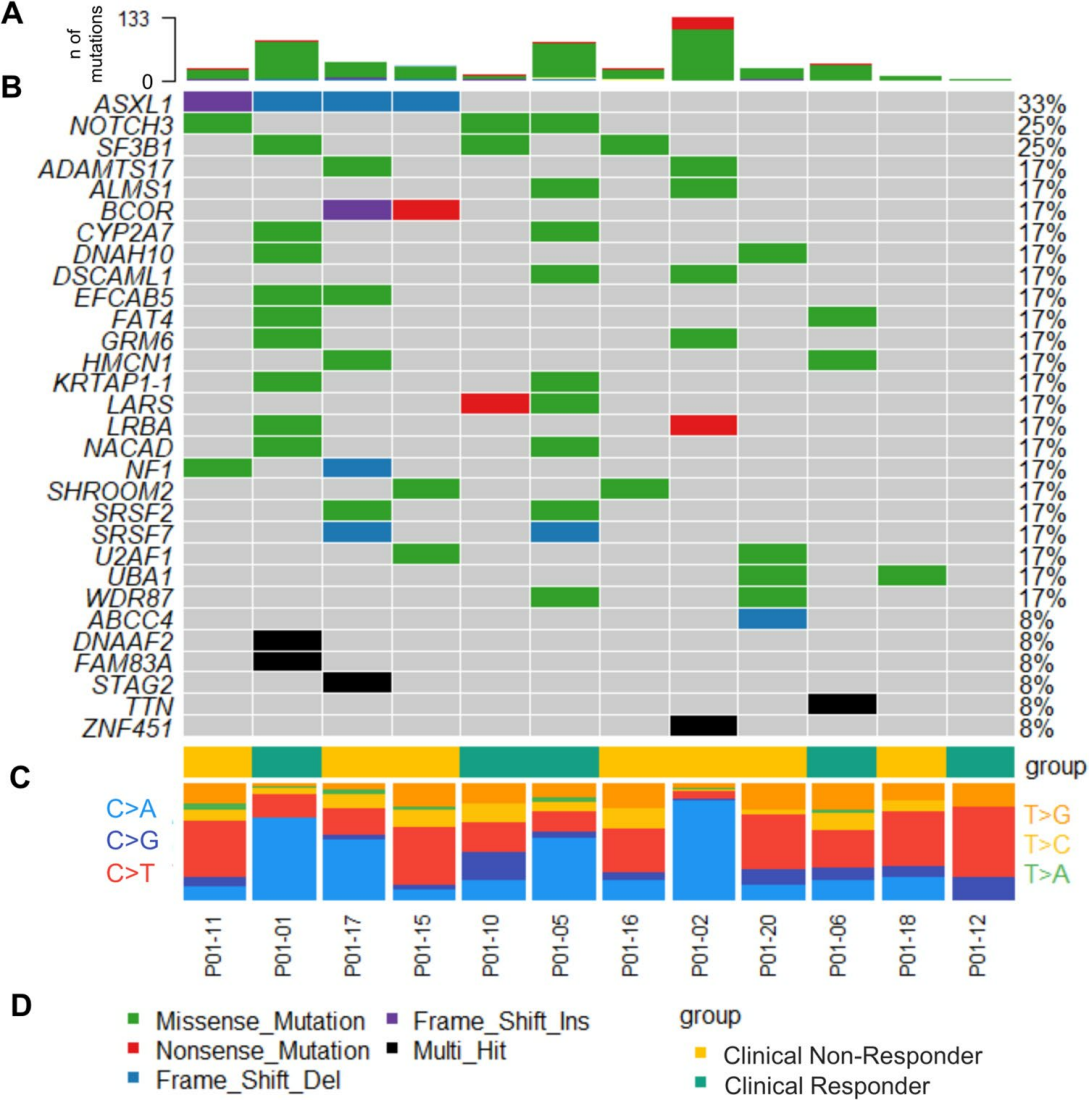
Mutational co-occurrence of the most frequently mutated genes. a Number of mutations per sample. **b** Oncoplot of the *n* = 30 most frequently detected mutations. **c** Group annotation for each sample. **d** nucleotide transitions for each sample. Patients P01-01, P01-02 and P01-05 show significantly more detected mutations and at the same time more C>A transitions

In Fig. 3, the co-occurrence of the *n* = 30 most frequently mutated genes at any time point is shown. Of note, patients P01-01, P01-02 and P01-05 showed markedly more mutations and more C>A transitions, possibly indicating a different mode of mutational acquisition.

Overall, there was no significant molecular difference between the previously classified clinical responders and non-responders.

### The apoptosis inhibitor Asunercept induces MDS clone size reduction subsequent to treatment

**Fig. 4:**
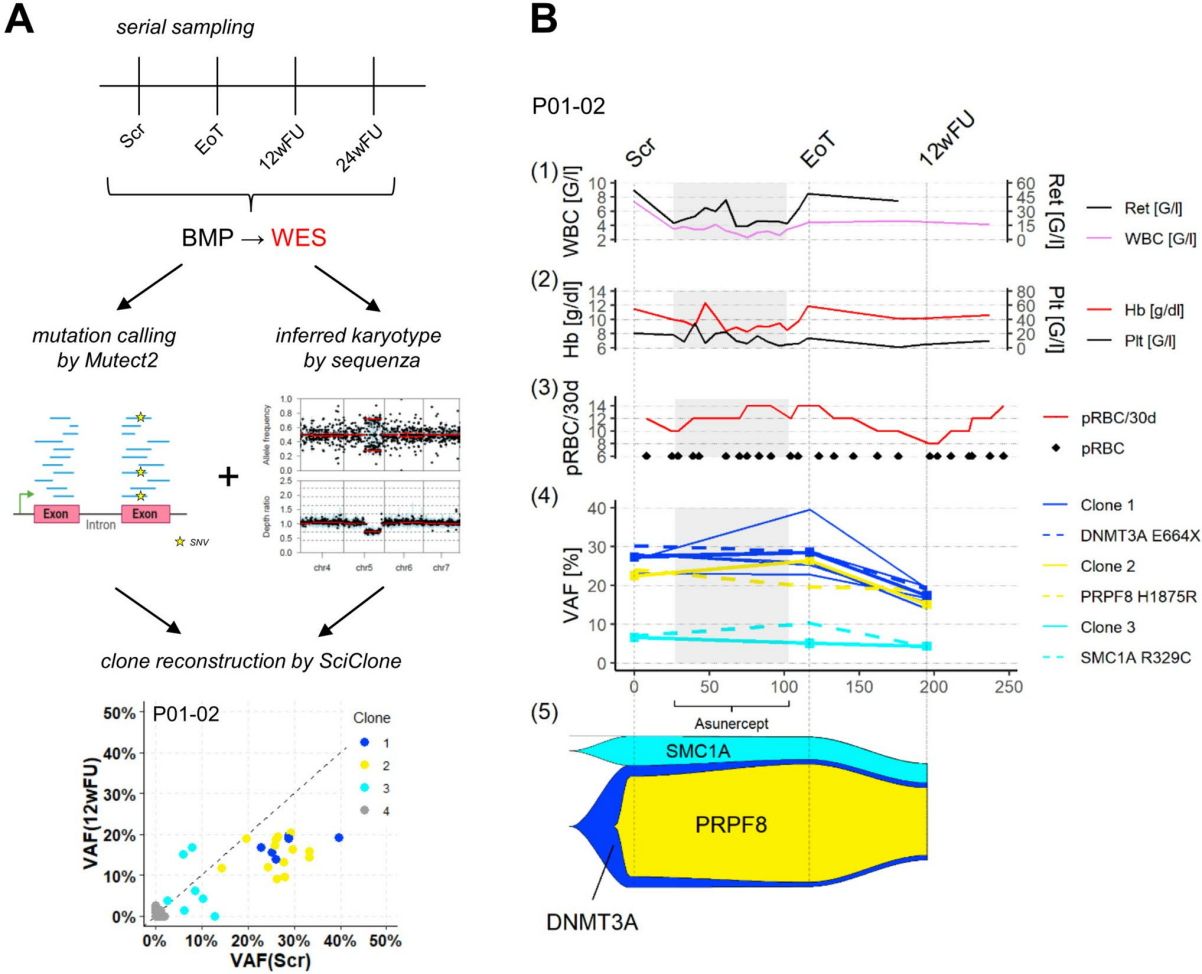
Assessment of clonal composition of the bone marrow in the course of time under treatment with Asunercept and correlation with clinical parameters. **a** Experimental setup. Serial sampling by a bone marrow puncture (BMP) was performed at the timepoints of Scr (screening), EoT (End of treatment), 12wFU (12 week follow-up) and 24 week follow-up (24wFU) and subjected to whole exome sequencing (WES). Mutation calling was performed by *Mutect2* [19], karyotype was inferred by *sequenza* [20], clones were reconstructed by *SciClone* [21], SNV: single nucleotide variant. **b** Within P01-02, Clone 1, with a *DNMT3A* p.E664X driver mutation, developed subclone 2, with a *PRPF8* p.H1875R driver mutation, and overgrew the bone marrow, displacing clone 4 (not shown). In correlation with clinical parameters an increase of Hb by approx. 2 g/dl was observed at the end of treatment (EoT) with no change in transfusion frequency (pRBC/30d). Moreover, consistent with the decrease in VAF of the dominant clone, transfusion frequency could be reduced by approximately 2 to 4 pRBCs in 30 days after treatment with stable hemoglobin values. (1) White blood cells (WBC) and absolute reticulocytes (Ret) in the course of time. (2) Hemoglobin (Hb) and platelet (Plt) levels in the course of time. (3) Administration of two packed red blood cell transfusions (pRBC) depictured as red line; amount of pRBC within 30 days (pRBC/30d). (4) VAF of mutated genes grouped by clone assessed with the tool SciClone, colored by functional group: blue = DNA methylation; yellow = splicing; turquoise = cohesion. (5) “fishplot” of the clonal composition of the bone marrow in the course of time

**Fig. 5:**
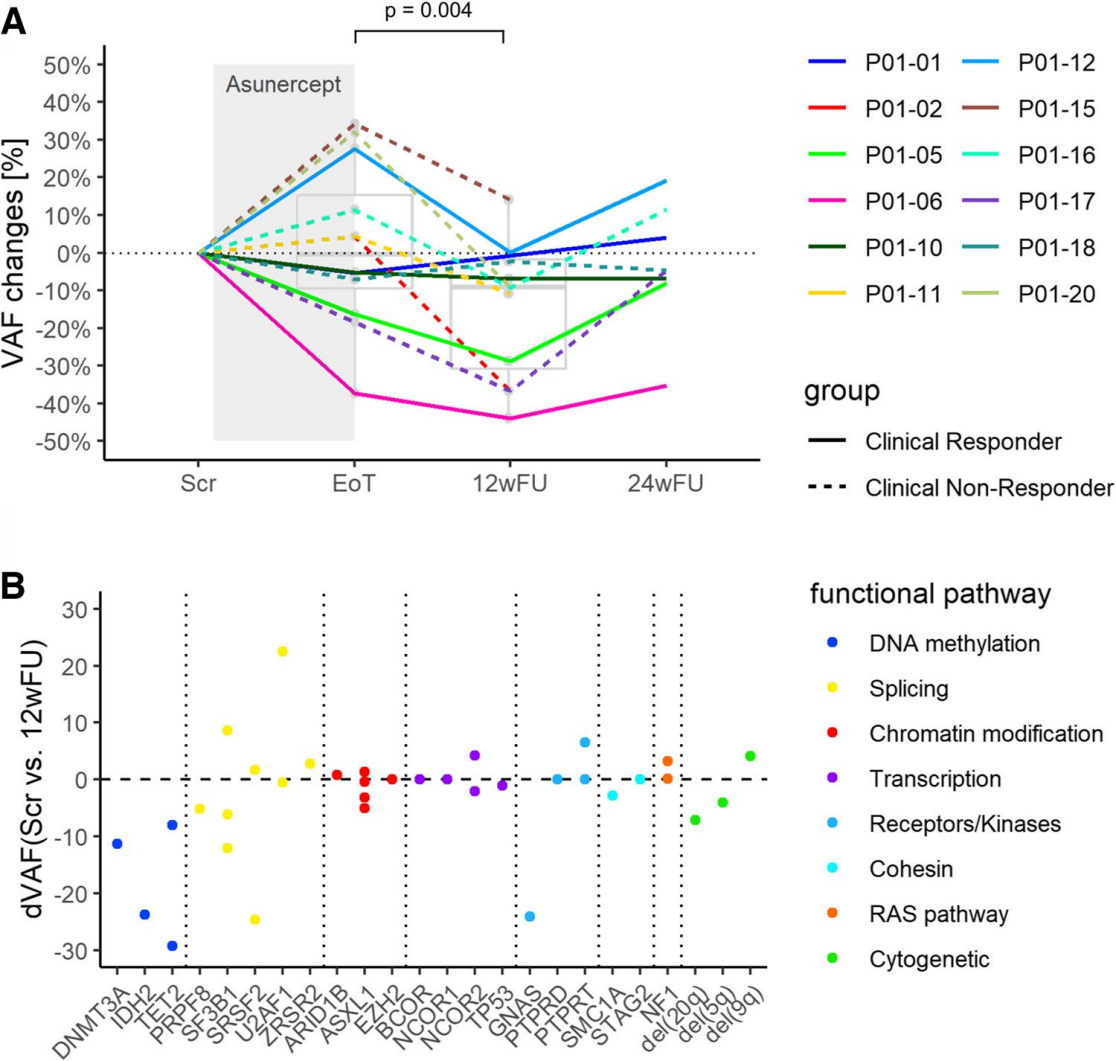
Clones carrying a mutation involved in methylation processes affected by Asunercept therapy. **a** VAF of dominant clones of all *n* = 12 patients over time, VAF changes as a relative change in percent. A marked decrease in VAF with a mean of 20% (range: 10.5–39.2%) between EoT and 12wFU in 9 of 12 (75%) patients was observed. For the significance testing, a paired T-test was used. **b** Delta VAF (dVAF) between Scr and 12wFU timepoint for all detected mutations of all *n* = 14 patients grouped by functional pathway. One point represents one mutation. *n* = 56 mutations showed a reduction of VAF by more than 10%, whereas only *n* = 23 mutations showed an increase of more than 10%. All *n* = 3 subclones from the patients 01-02, 01-05 and 01-17, which were driven by a mutation involved in methylation regulation (*DNMT3A* *n* = 1, *IDH2* *n* = 1, *TET2* *n* = 1; P01-17 carried two *TET2* Mutations), were responsive on a molecular level

In order to identify distinct subclones, which are sensitive to Asunercept therapy, we reconstructed the clonal composition in the bone marrow using *SciClone* by VAF alterations under therapy and in the course of time (Fig. 4a). In addition to mutational data from exome sequencing, copy number variation (CNV) data was accounted for. These were available from routine cytogenetic assessment and additionally, CNVs were assessed from single nucleotide polymorphisms (SNP) allelic ratios inferred from the WES data using *Sequenza* (Supplemental Fig. 1). We could identify an average of 3.5 separable clones per patient (range 2–6). In addition, we were able to reconstruct the most likely hierarchical relationship between the clones in *n* = 3 cases (P01-02, P01-05, P01-17). The same principles as already published in [14] were applied (Supplemental methods). In the remaining cases, mutational VAF changes were not discriminative enough to unequivocally determine their relative order of historic acquisition. Based on the serial WES data, we were able to generate time course plots of mutational variant allele frequencies (VAF) and clinical parameters such as white blood cell count (WBC), hemoglobin levels (Hb), frequency of red blood concentrate (RBC) transfusions and when applicable, platelet concentrates (PC) in correlation to treatment with Asunercept. In the case of P01-02, this patient showed a putative molecular response demonstrated by a decrease in the *DNMT3A*-driven clone including the *PRPF8*-driven subclone following treatment while the *SMC1A*-driven clone remained stable. This coincided with the clinical findings that hemoglobin (Hb) was increased and transfused pRBCs per 30 days were reduced (Fig. 4b). Also in other cases, we observed that while the VAFs of clone-defining mutations remained stable or even increased during the actual treatment duration with Asunercept, they were considerably reduced post treatment in relation to the baseline of End of treatment (EoT) in the 12-week follow-up period (12wFU) (Fig. 5a). In total, the observation of significant VAF reduction of the dominant clone post treatment with Asunercept relative to the baseline of EoT during the 12wFU period by at least 10% was found in 9 out of 12 cases (P01-02, P01-05, P01-06, P01-11, P01-12, P01-15, P01-16, P01-17, P01-20; mean reduction 20%, range: 10.5–39.2%; Fig. 5a, Supplementary Figs. 2–12).

Additionally, we calculated the delta VAF as an absolute change between the Scr and 12wFU timepoint to detect early molecular responders **(**mean −3.1; range −29.2 to 22.5; Fig. 5b**)**. Interestingly, all *n* = 3 patients harboring a mutation involved in methylation processes showed an early (P01-05, P01-17) and profound (P01-02) molecular response already during the 12-week treatment period (Figs. 4a, 5a, b and Supplemental Figs. 3, 10). In *n* = 2 of these cases (P01-02, P01-05, Fig. 4a, Supplemental Fig. 3) this reduction of the dominant clonal VAFs was associated with a reduction of transfused pRBC per 30 days by at least 4 units over 30 days.

For the remaining case (P01-17), an increase in pRBCs transfusions was observed. However, this patient developed sepsis during treatment with Asunercept, therefore the clinical response might be skewed and is hence not evaluable.

All clinical findings and molecular results are summarized in Table 3.

**Table 3:** Comparison of clinical results and molecular findings

Patient	Clinical findings	Molecular results
P01-01	Clinical responder	Stable molecular BM composition
P01-02	Clinical non-responder, but transient Hb increase and pRBCs need reduction	Profound VAF reduction of dominant clone; mutation involved in methylation processes
P01-05	Clinical responder	Early VAF reduction of dominant clone; mutation involved in methylation processes
P01-06	Clinical responder	Early VAF reduction of dominant clone
P01-10	Clinical responder	Stable molecular BM composition
P01-11	Clinical non-responder	VAF reduction of dominant clone
P01-12	Clinical responder	VAF reduction of dominant clone
P01-15	Clinical non-responder	VAF reduction of dominant clone
P01-16	Clinical non-responder	VAF reduction of dominant clone
P01-17	Clinical non-responder, clinical response not evaluable due to sepsis	Early VAF reduction of dominant clone; mutation involved in methylation processes
P01-18	Clinical non-responder	Stable molecular BM composition
P01-20	Clinical non-responder	VAF reduction of dominant clone

### Asunercept does not lead to clonal expansion and progression in MDS

In the preceding clinical phase I study it was already shown that there was no evidence that Asunercept would promote progression to secondary AML on a clinical level. In line with this, none of the *n* = 12 patients of the current study displayed a significant increase in VAF at the molecular level (Supplemental Figs. 2–12). Even clones that carried a prognostically unfavorable mutation, such as P01-16 with a TP53 p.V173M mutation, showed no expansion in the bone marrow during treatment and follow-up with Asunercept (Supplemental Fig. 9).

As previously stated, in the original publication *n* = 2 patients had been described with leukemic progression, reflecting a typical course of the disease in an unfavorable risk constellation. Of these, *n* = 1 patient, P01-15, was further analyzed on a molecular level in this study. Again, we observed no increase of the VAF of any mutation (Supplemental Fig. 8).

In P01-17, sepsis occurred as a complication after 8 doses of Asunercept, which resulted in termination of therapy and granulocyte colony stimulating factor (G-CSF) was applied prophylactically during neutropenia. At the following study visit, an increased blast count from previously below 2% to 5% was observed, which was attributed to the application of G-CSF. At the same time point molecular genetics even showed a decrease of the dominant clone (Supplemental Fig. 10). Furthermore, in this patient, clonal evolution was observed at the 24-week follow-up. Whether this is due to the natural progression of the disease, the administration of GCS-F, or the Asunercept therapy remains unclear.

## Discussion

In lower risk MDS, only few treatment options are available to sufficiently treat anemia.

However, over the last years, it has become more and more obvious that stratification of patients based on genetic profiles could help identifying better individual therapies [27]. In this context, the most notable successes are the use of Luspatercept in MDS with SF3B1 mutations and Lenalidomide in MDS patients with 5q deletion [2, 28]. In contrast, there is no specific therapy available for the majority of lower risk patients without these specific lesions.

In our study, we performed a comprehensive molecular analysis of *n* = 12 patients who have been exome sequenced at *n* = 58 time points from a clinical trial with the novel compound Asunercept. While the number of analyzed samples was too small to draw robust conclusions, we were able to show a methodological proof of principle that we mutations in methylation associated genes that showed marked reduction of VAF following the clinical treatment with Asunercept.

Although limited clinical responses were observed in the preceding clinical trial [13], our results indicate that a majority of patients showed a change in the bone marrow compartment in terms of a decrease of the dominant clone by more than 10% VAF after therapy.

Overall, the mutational landscape in patient samples of our study was similar to other large studies investigating the mutational spectrum of MDS [26].

In accordance with the preceding clinical trial and the therein established definition, patients with a continuous reduction in transfusion burden were defined as “clinical responders”. General molecular characterization in terms of mutated genes or affected pathways of our sequencing cohort revealed no significant difference between these clinical responders and non-responders.

Through more sophisticated analysis and the reconstruction of specific subclones, we were able to observe more subtle effects of Asunercept therapy. We found that the effect of Asunercept occurs mainly after the 12-weeks therapy and leads to a reduction of the dominant clone during the period of 12wFU. Based on that result, molecular response was defined in this study as a relative reduction in VAF of the dominant clone by more than 10% between EoT and 12wFU and was observed in 9 out of 12 cases (75%). A possible explanation for this delayed therapeutic response could be that blocking CD95 and its ligand allows immature erythroid cells to mature and finally differentiate [11]. Why this also results in a decrease in the proportion of mutated cells remains unclear. We hypothesize that the proportion of healthy, non-diseased erythropoiesis increases through maturation, thus causing a relative reduction in the allelic burden from mutated cells. From this observation, we hypothesize that a longer duration of therapy could possibly be beneficial.

Interestingly, all *n* = 3 patients (P01-02, P01-05, P01-17) harboring a mutation involved in methylation processes showed an early and profound reduction of VAF already during the 12-week treatment period, which was translated into a reduced transfusion burden in *n* = 2 cases.

In conclusion, by reconstruction of mutational hierarchies of serial exome sequenced samples, there appears to be an effect of Asunercept treatment on the bone marrow, in the terms of a reduction in clone size, which only occurs after a longer treatment period of around 12 weeks.

It was recently demonstrated that Asunercept showed good efficacy in the treatment of glioblastoma [29, 30]. Although CD95 signaling may be relevant for multiple aspects of tumor growth, the mechanism of action of Asunercept in glioblastoma remains unclear. However, CD95L promoter methylation in Glioblastoma is discussed as a biomarker for therapy response [31]. To verify whether this is also applicable to MDS, the methylation of the CD95L promoter was examined, but no correlation between responders and non-responders could be observed (data not shown).

Asunercept appears to be a safe drug with a low spectrum of side effects. Although apoptosis as a hallmark of cancer [32] is abolished by the inhibitor [33], no progression was observable at the clinical and molecular genetic level. With our analyses, we demonstrated a stable clonal composition without development of aggressive subclones and with no increased risk of progression to secondary AML.

Using this approach, we identified a subset of patients carrying specific genetic aberrations in genes regulating methylation processes such as DNMT3A, TET2 and IDH2, which may be a cue for further optimizing therapy with Asunercept.

This underscores the need for more detailed and accompanying broad NGS based molecular monitoring of MDS patients under treatment in order to improve understanding of molecular responses and mechanisms of therapeutic agents in MDS.

### Supplementary Information

Below is the link to the electronic supplementary material.

ESM 1 (DOCX 39 KB)

Supplementary Figure 1 Chromosomal copy number variation assessed by sequenza. A: VAF of single nucleotide polymorphisms (SNPs). B: Ratio of sequencing depth C: Assessed copy number out of the data of A and B. D: karyotype assessed by routine cytogenetics. (JPG 683 KB)

Supplementary Figure 2-12 Clonal composition of all n=11 patients (without P01-02) in the course of time under therapy with Asunercept and in correlation with clinical parameters. (1) White blood cells (WBC) and absolute reticulocytes (Ret) in the course of time. (2) Hemoglobin (Hb) and platelet (Plt) levels in the course of time. (3) Administration of two packed red blood cell transfusions (pRBC) depictured as red line; amount of pRBC within 30 days (pRBC/30d). (4) VAF of mutated genes grouped by clone assessed with the tool SciClone, colored by functional group: blue = DNA methylation; yellow = splicing; turquoise = cohesion; purple = transcription; pink = DNA repair/cell cycle; green = cytogenetic changes; red = chromatin modification; orange = RAS pathway; grey = no driver mutation; gold/grey = no driver mutation; blue/grey = no driver mutation; pink/grey = no driver mutation; (5) “fishplot” of the clonal composition of the bone marrow in the course of time. (JPG 960 KB)

(JPG 385 KB)

(JPG 314 KB)

(JPG 316 KB)

(JPG 386 KB)

(JPG 282 KB)

(JPG 351 KB)

(JPG 329 KB)

(JPG 376 KB)

(JPG 306 KB)

(JPG 298 KB)

## Data Availability

No datasets were generated or analysed during the current study.
