# Supplementary material for: Treatment with the apoptosis inhibitor Asunercept reduces clone sizes in patients with lower risk Myelodysplastic Neoplasms
Source: Ann Hematol. 2024 Feb 27;103(4):1221–33. doi: 10.1007/s00277-024-05664-5 (PMC10940491; doi:10.1007/s00277-024-05664-5)
Supplement: Supplementary file 1 — (DOCX 39 KB) [file 277_2024_5664_MOESM1_ESM.docx]

Supplemental methods

Reconstruction of clonal hierarchies

To make assumptions about the most likely clonal composition, the following criteria were applied as already published in [14]:

1. In order to consider mutation “A” being acquired earlier than mutation “B” we required that at least one investigated sample was requested to show a higher burden for mutation “A” and a difference in frequency of 10% relative to mutation “B” or
2. at least 3 investigated samples were requested to exhibit a higher mutational frequency for mutation “A” and a difference in frequency of 5% relative to mutation “B”.
3. Two clones were considered mutually exclusive if the sum of the allelic frequency of both heterozygous mutations exceed 55% (theoretically 51% would be sufficient) in at least 1 sample to be confident, that the clones did not arose within the same cell.

In addition, the following criteria, based on own assumptions, were applied:

1. Simultaneous VAF changes of at least 5% (e.g., under therapy) with less than 5% divergence between clone "A" and subclone "B" were considered to indicate that the two clones originated from the same cell.
2. When a definitive determination of the clonal hierarchy was elusive, we illustrated the likeliest composition by observing simultaneous VAF shifts over time.
