# Supplementary figures and images for: Treatment with the apoptosis inhibitor Asunercept reduces clone sizes in patients with lower risk Myelodysplastic Neoplasms
Source: Ann Hematol. 2024 Feb 27;103(4):1221–33. doi: 10.1007/s00277-024-05664-5 (PMC10940491; doi:10.1007/s00277-024-05664-5)

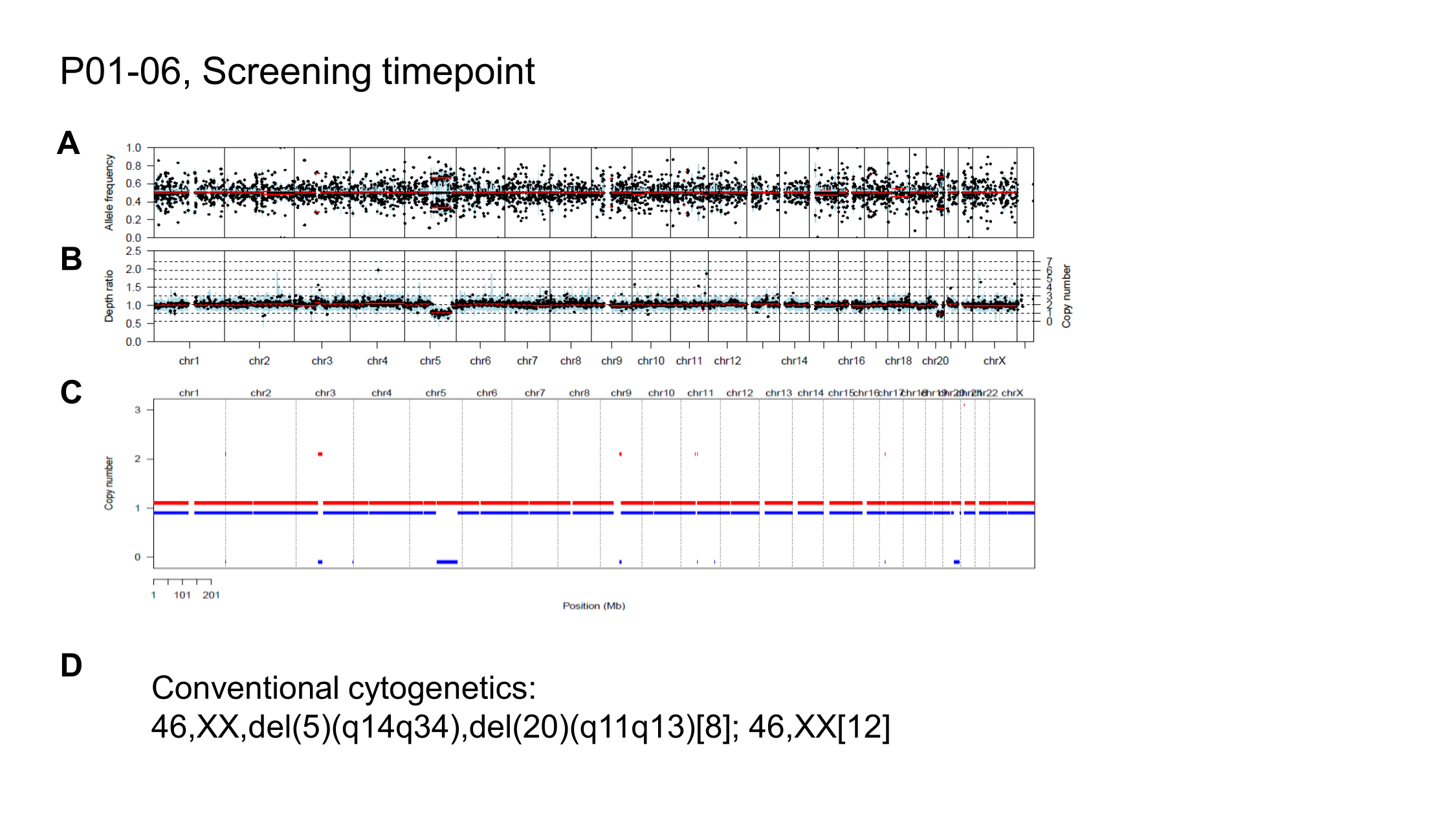

Supplement: Supplementary file 2 — Chromosomal copy number variation assessed by sequenza. A: VAF of single nucleotide polymorphisms (SNPs). B: Ratio of sequencing depth C: Assessed copy number out of the data of A and B. D: karyotype assessed by routine cytogenetics. (JPG 683 KB) [file 277_2024_5664_MOESM2_ESM.jpg]

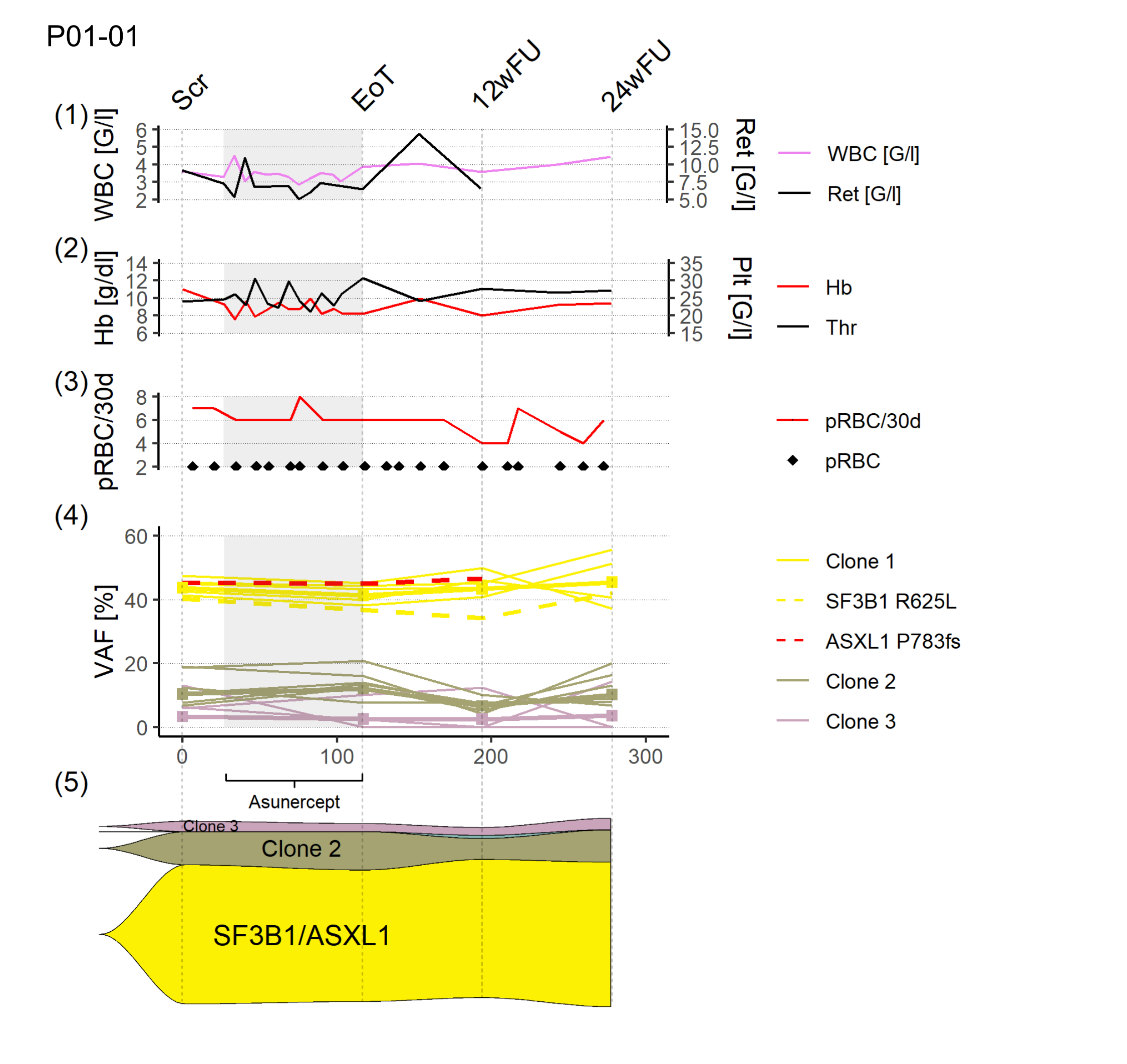

Supplement: Supplementary file 3 — Clonal composition of all n=11 patients (without P01-02) in the course of time under therapy with Asunercept and in correlation with clinical parameters. (1) White blood cells (WBC) and absolute reticulocytes (Ret) in the course of time. (2) Hemoglobin (Hb) and platelet (Plt) levels in the course of time. (3) Administration of two packed red blood cell transfusions (pRBC) depictured as red line; amount of pRBC within 30 days (pRBC/30d). (4) VAF of mutated genes grouped by clone assessed with the tool SciClone, colored by functional group: blue = DNA methylation; yellow = splicing; turquoise = cohesion; purple = transcription; pink = DNA repair/cell cycle; green = cytogenetic changes; red = chromatin modification; orange = RAS pathway; grey = no driver mutation; gold/grey = no driver mutation; blue/grey = no driver mutation; pink/grey = no driver mutation; (5) “fishplot” of the clonal composition of the bone marrow in the course of time. (JPG 960 KB) [file 277_2024_5664_MOESM3_ESM.jpg]

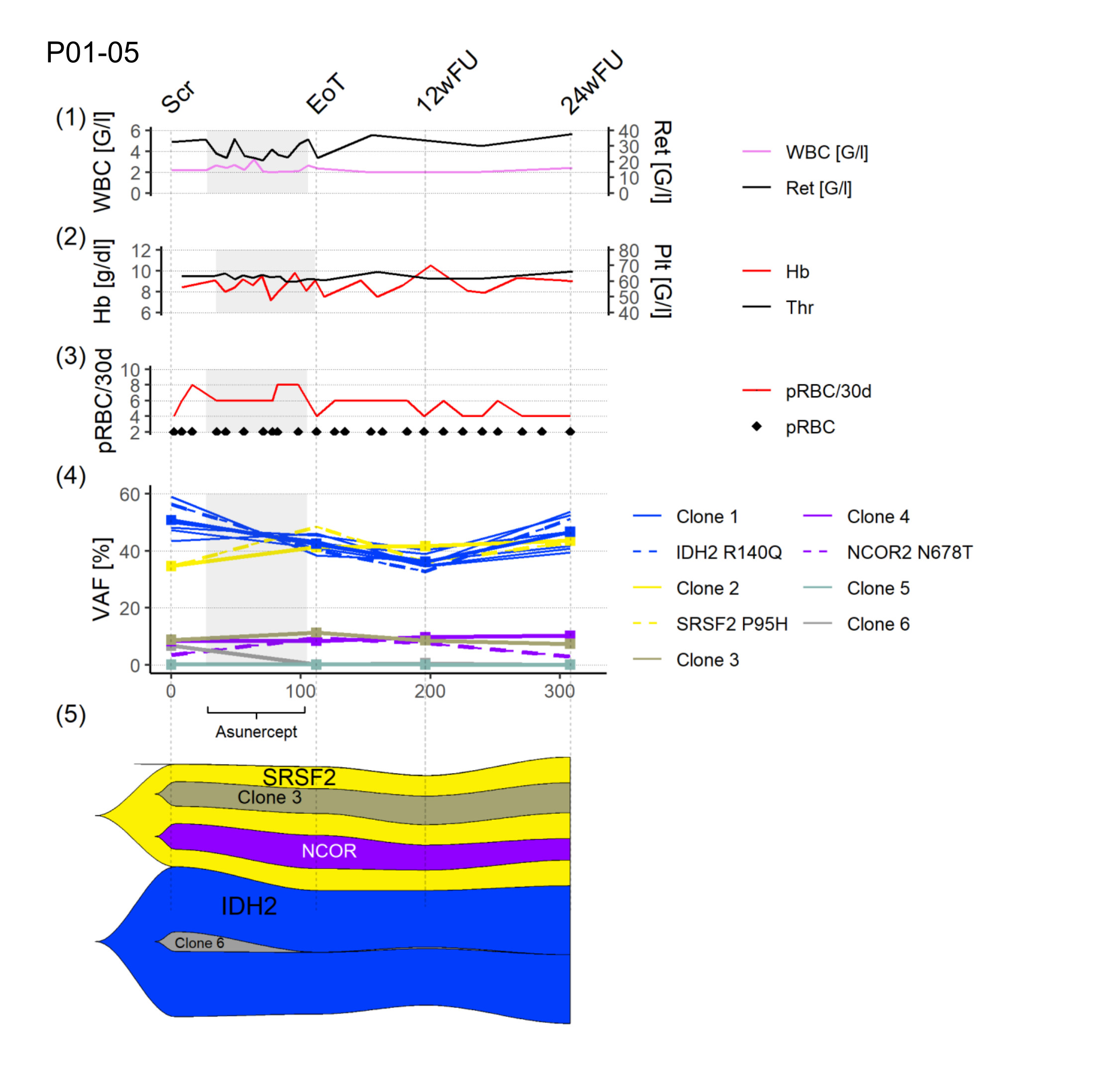

Supplement: Supplementary file 4 — (JPG 385 KB) [file 277_2024_5664_MOESM4_ESM.jpg]

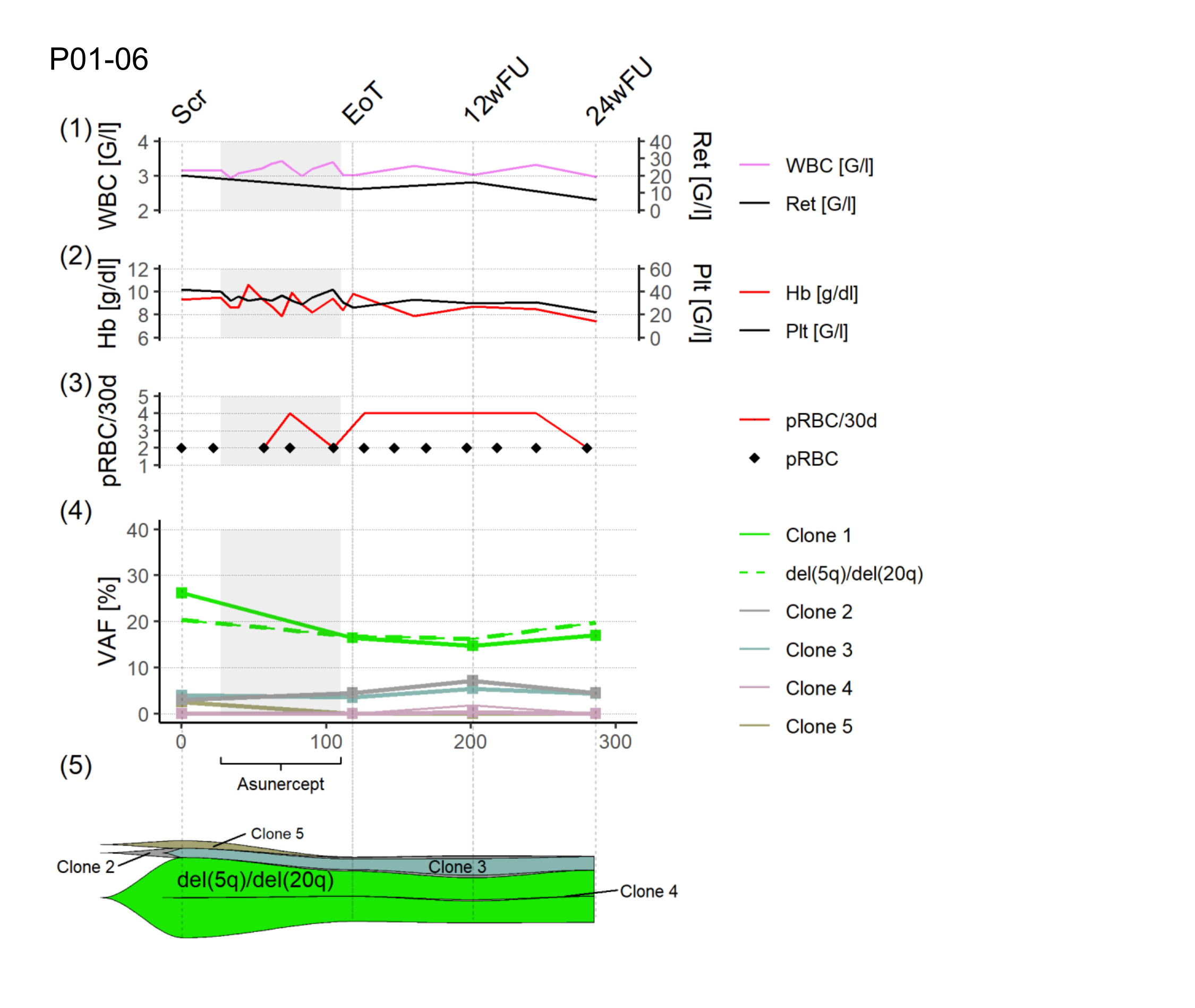

Supplement: Supplementary file 5 — (JPG 314 KB) [file 277_2024_5664_MOESM5_ESM.jpg]

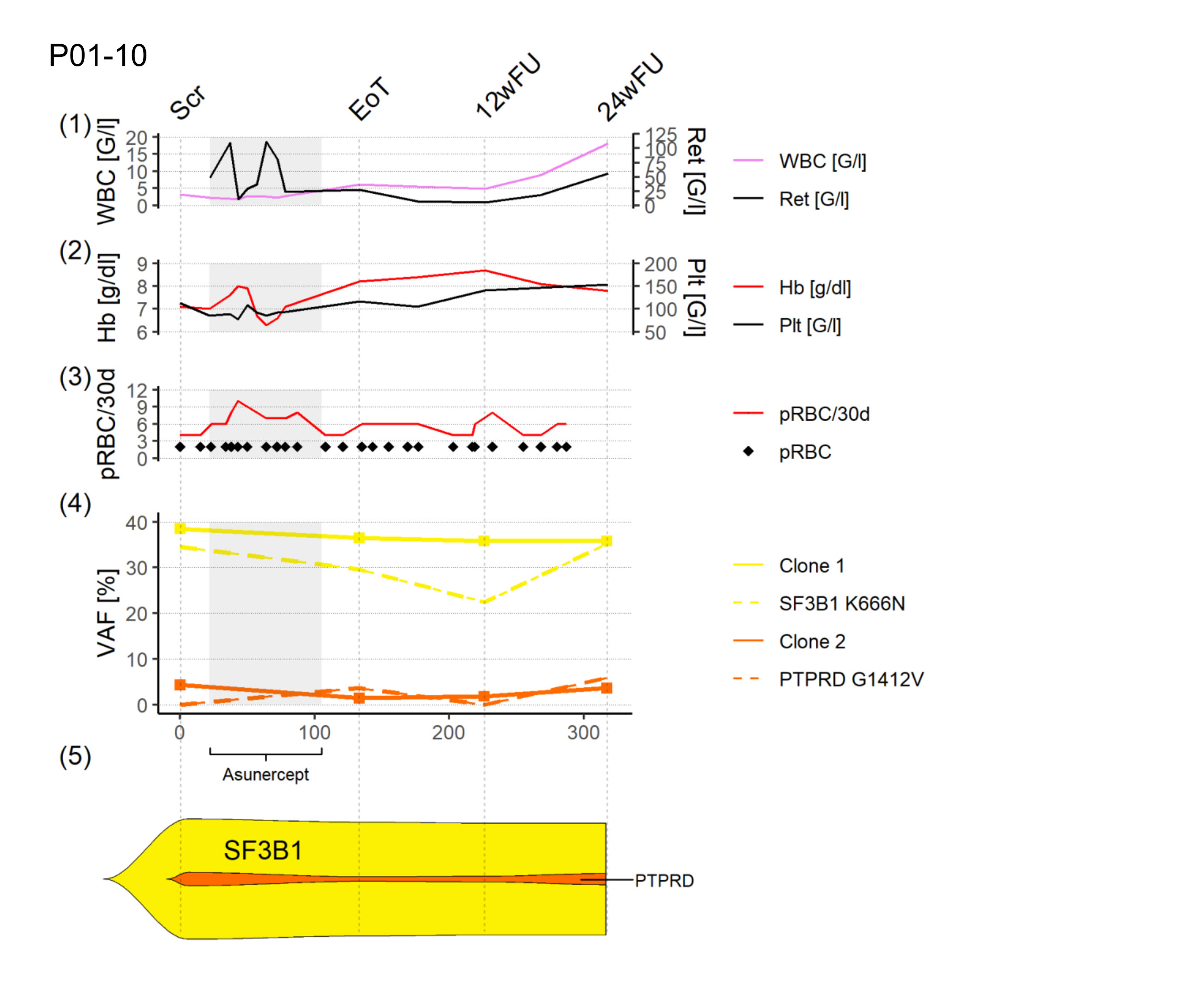

Supplement: Supplementary file 6 — (JPG 316 KB) [file 277_2024_5664_MOESM6_ESM.jpg]

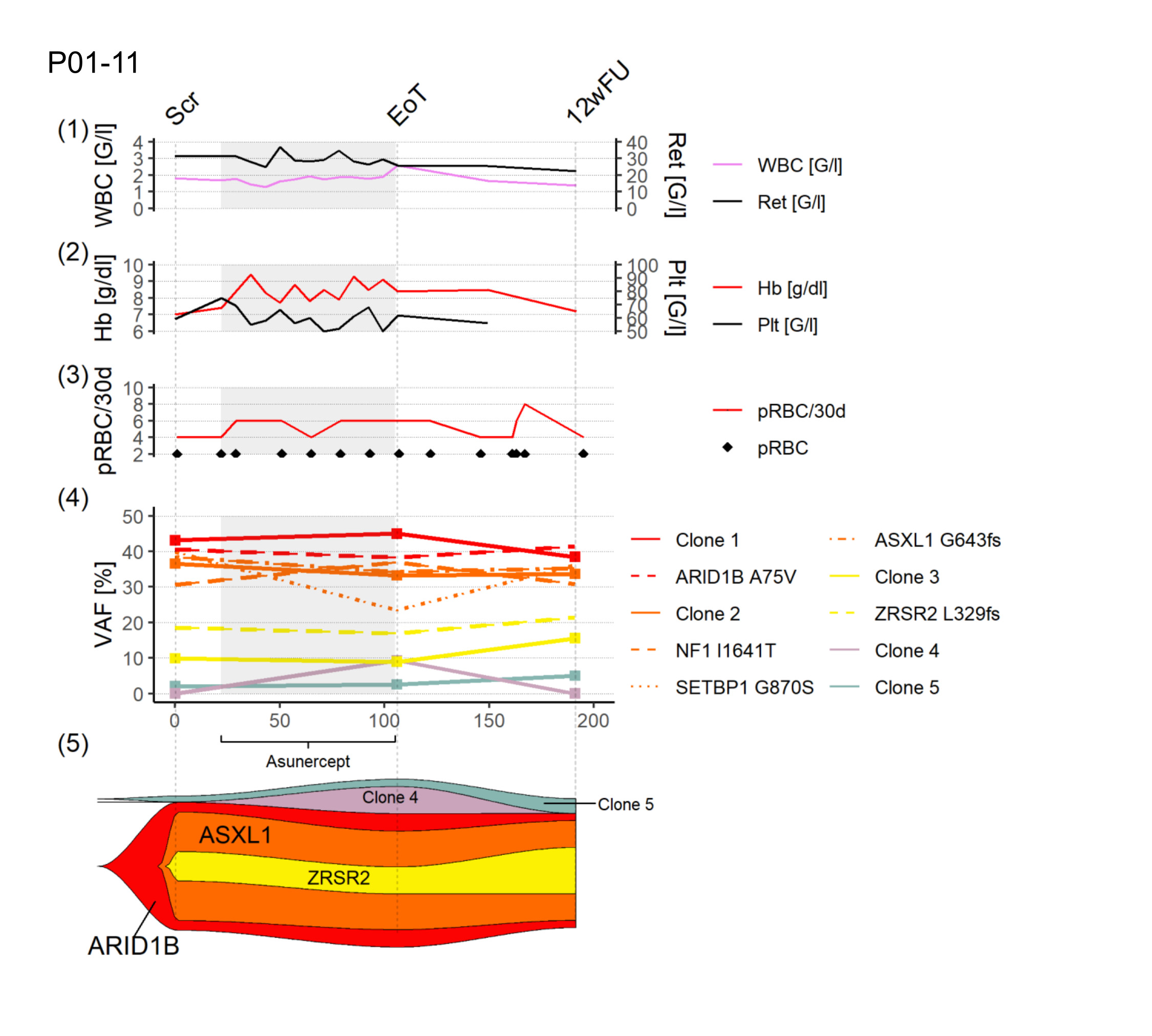

Supplement: Supplementary file 7 — (JPG 386 KB) [file 277_2024_5664_MOESM7_ESM.jpg]

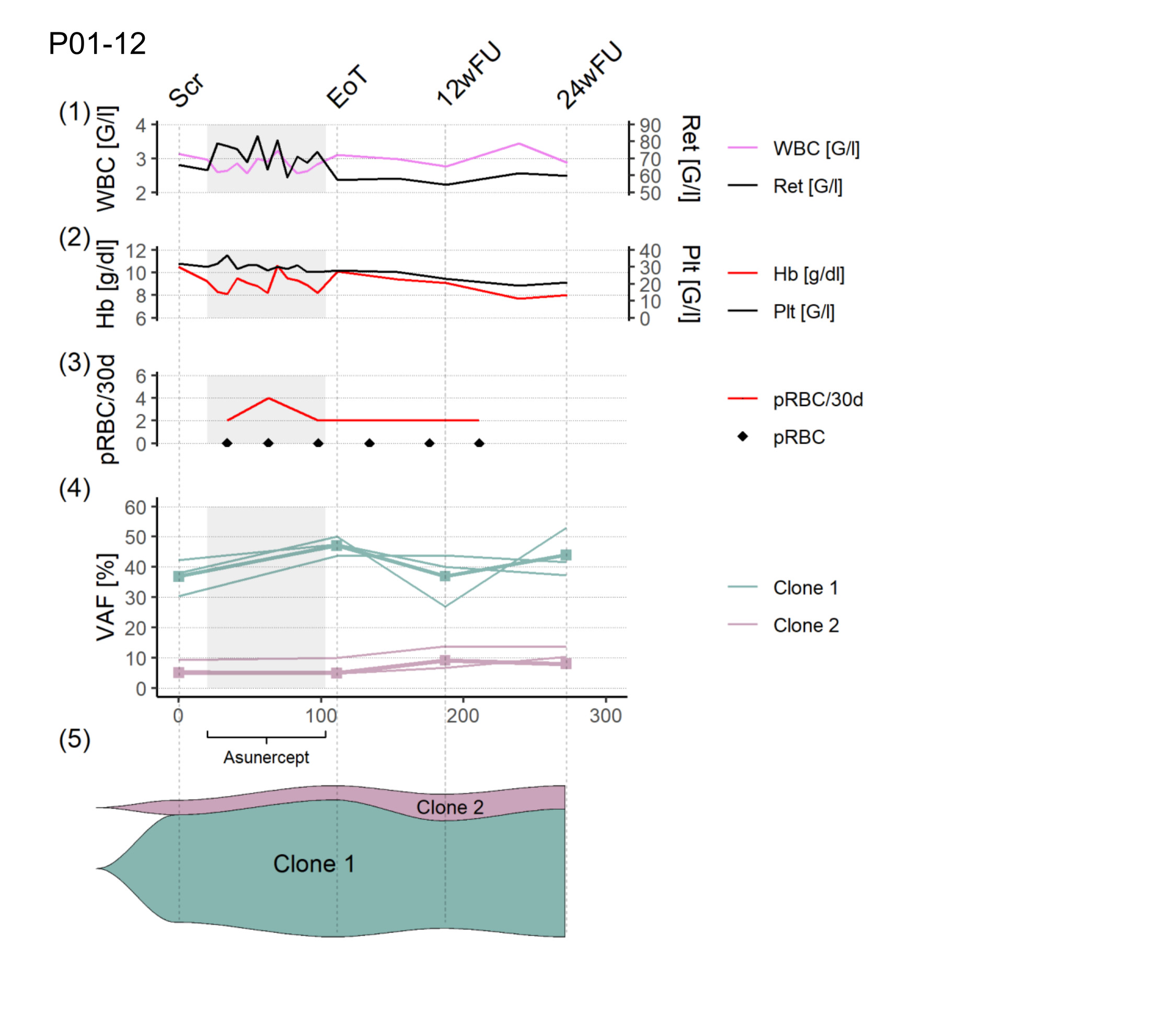

Supplement: Supplementary file 8 — (JPG 282 KB) [file 277_2024_5664_MOESM8_ESM.jpg]

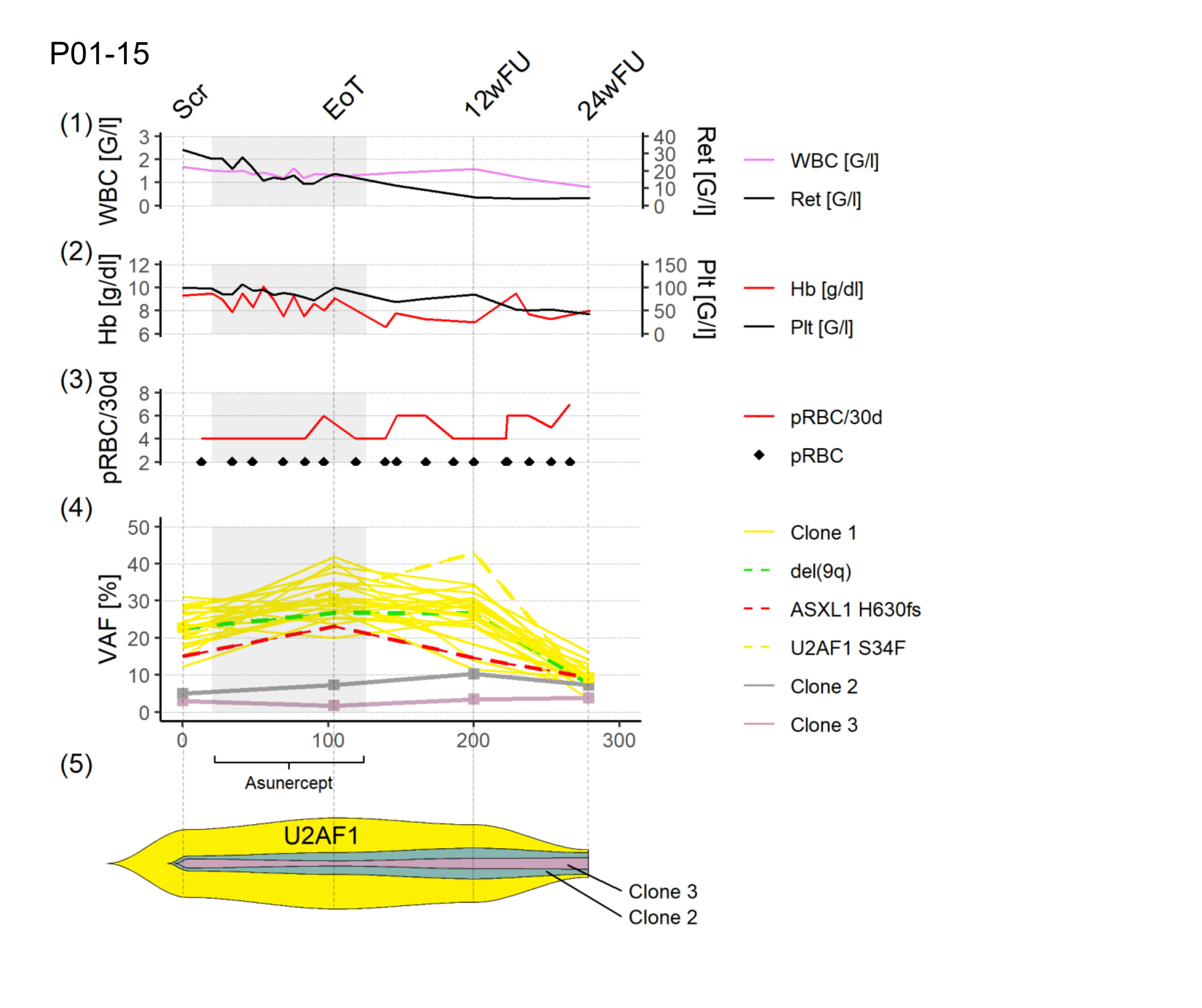

Supplement: Supplementary file 9 — (JPG 351 KB) [file 277_2024_5664_MOESM9_ESM.jpg]

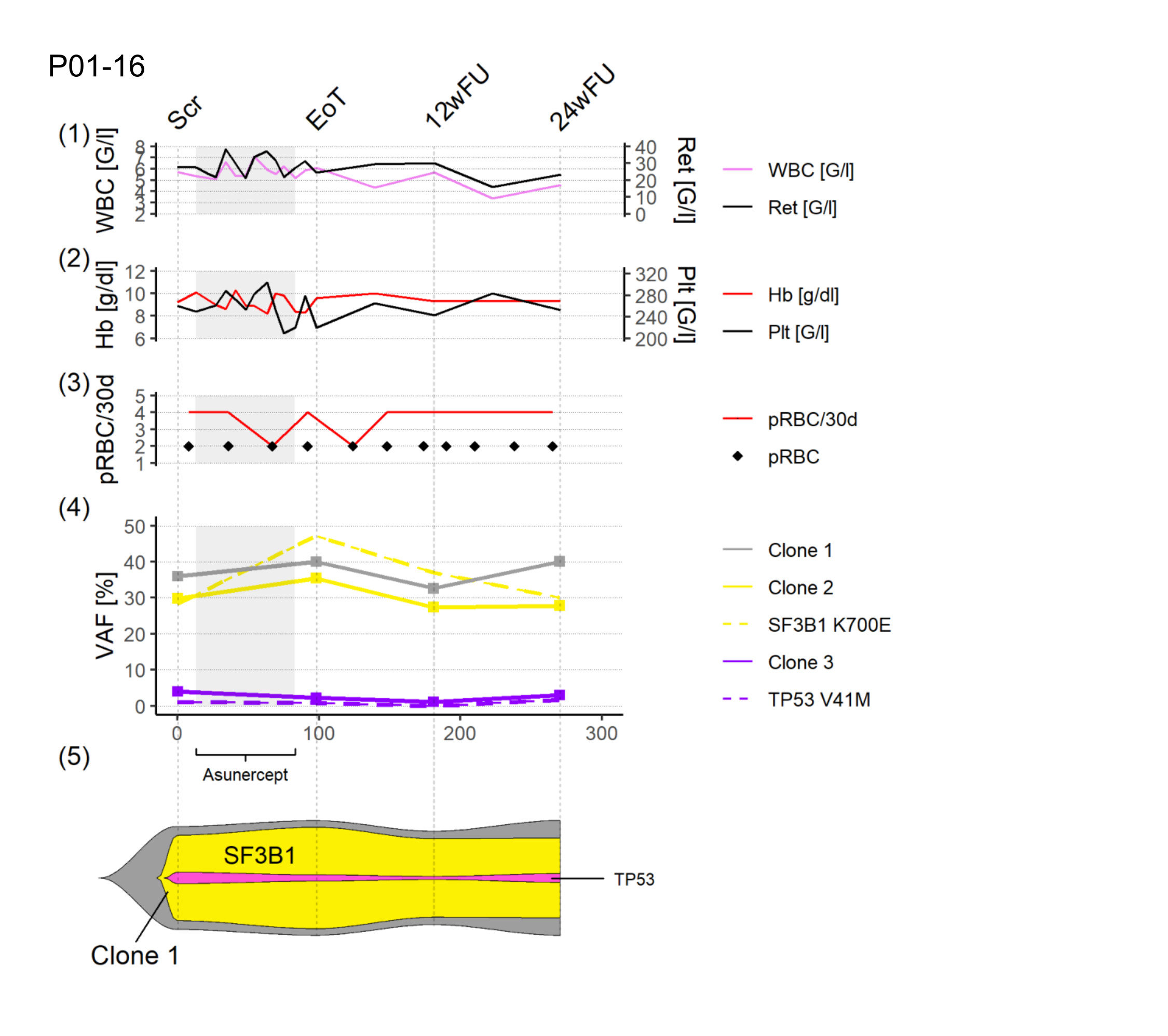

Supplement: Supplementary file 10 — (JPG 329 KB) [file 277_2024_5664_MOESM10_ESM.jpg]

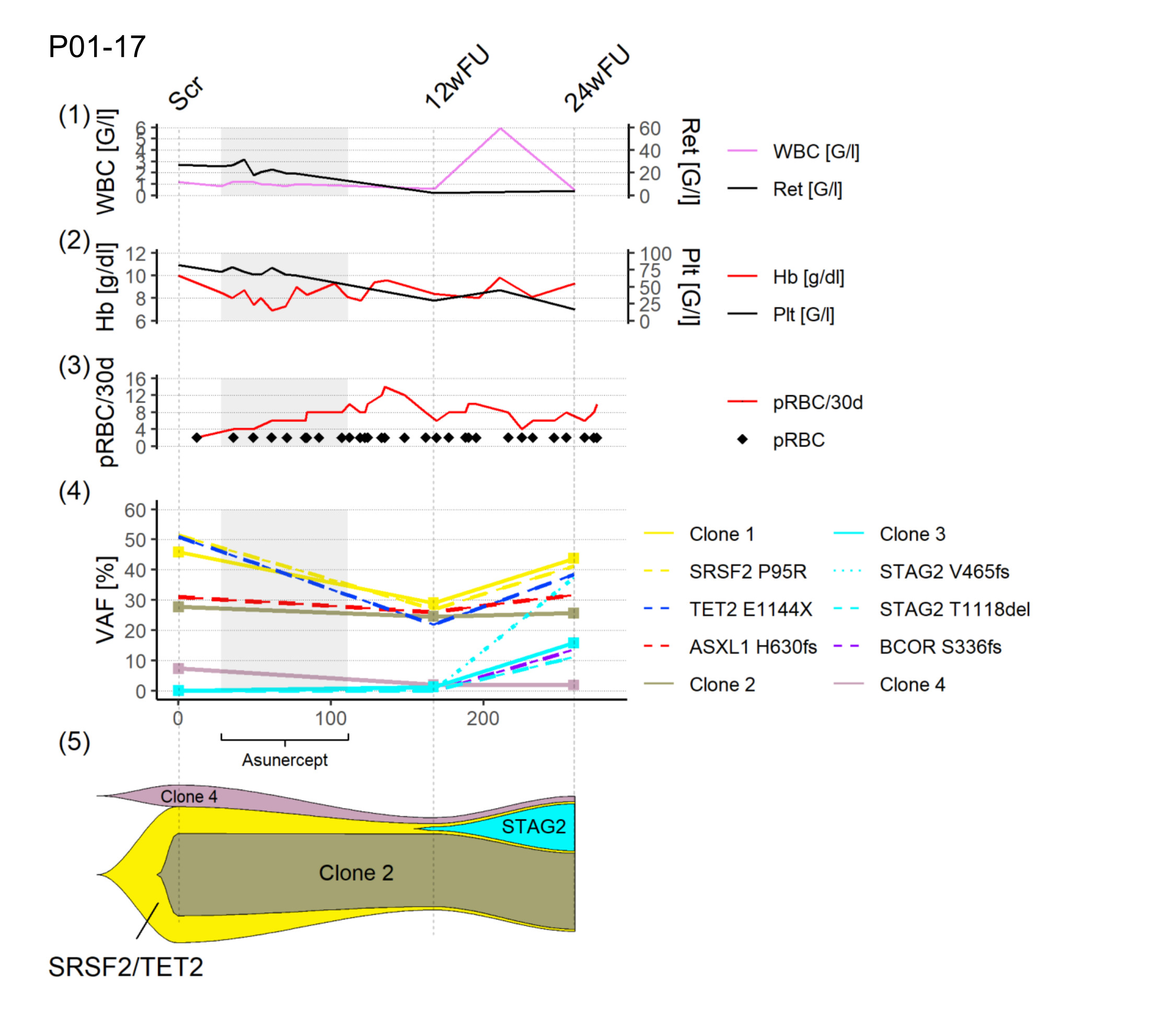

Supplement: Supplementary file 11 — (JPG 376 KB) [file 277_2024_5664_MOESM11_ESM.jpg]

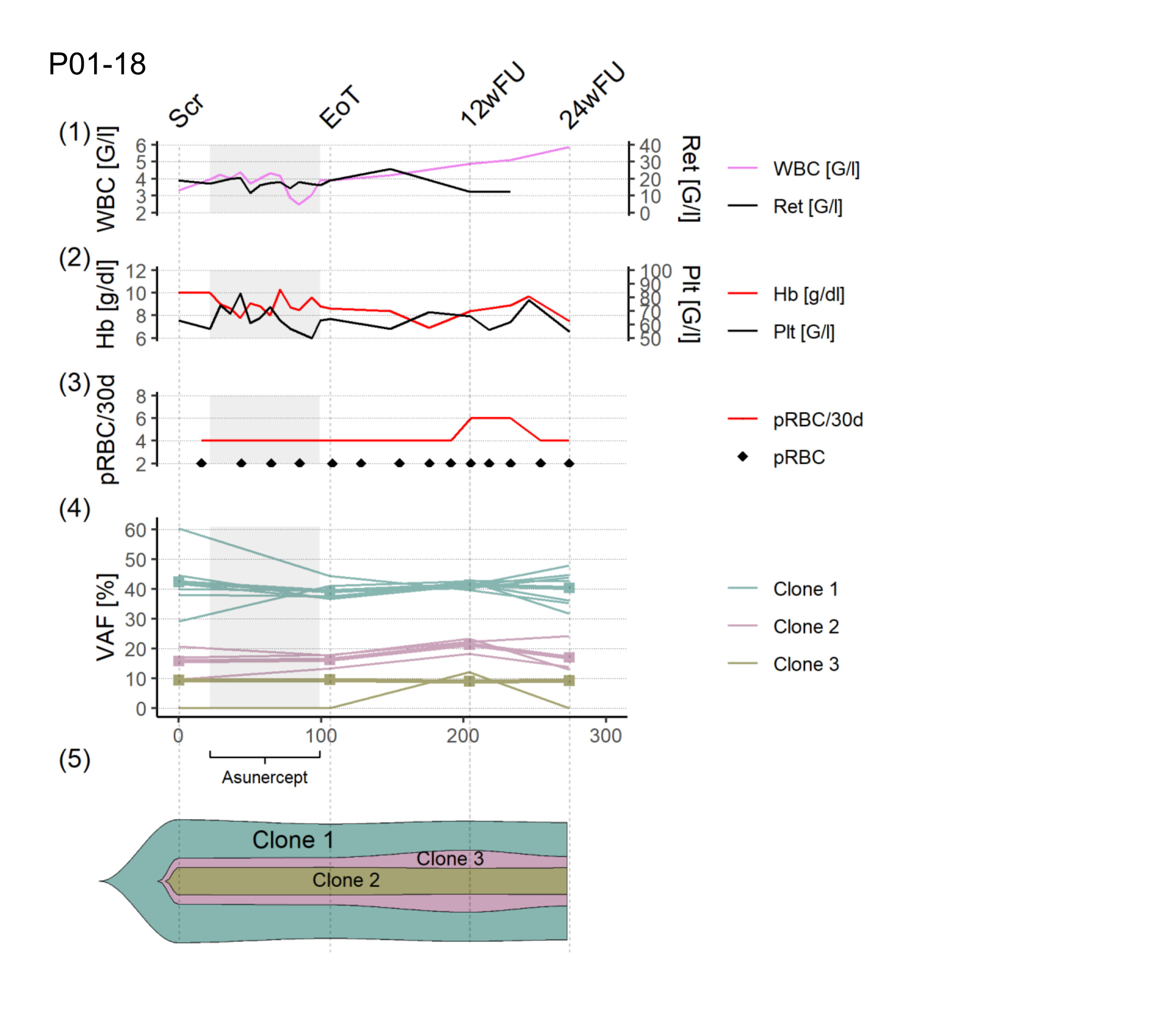

Supplement: Supplementary file 12 — (JPG 306 KB) [file 277_2024_5664_MOESM12_ESM.jpg]

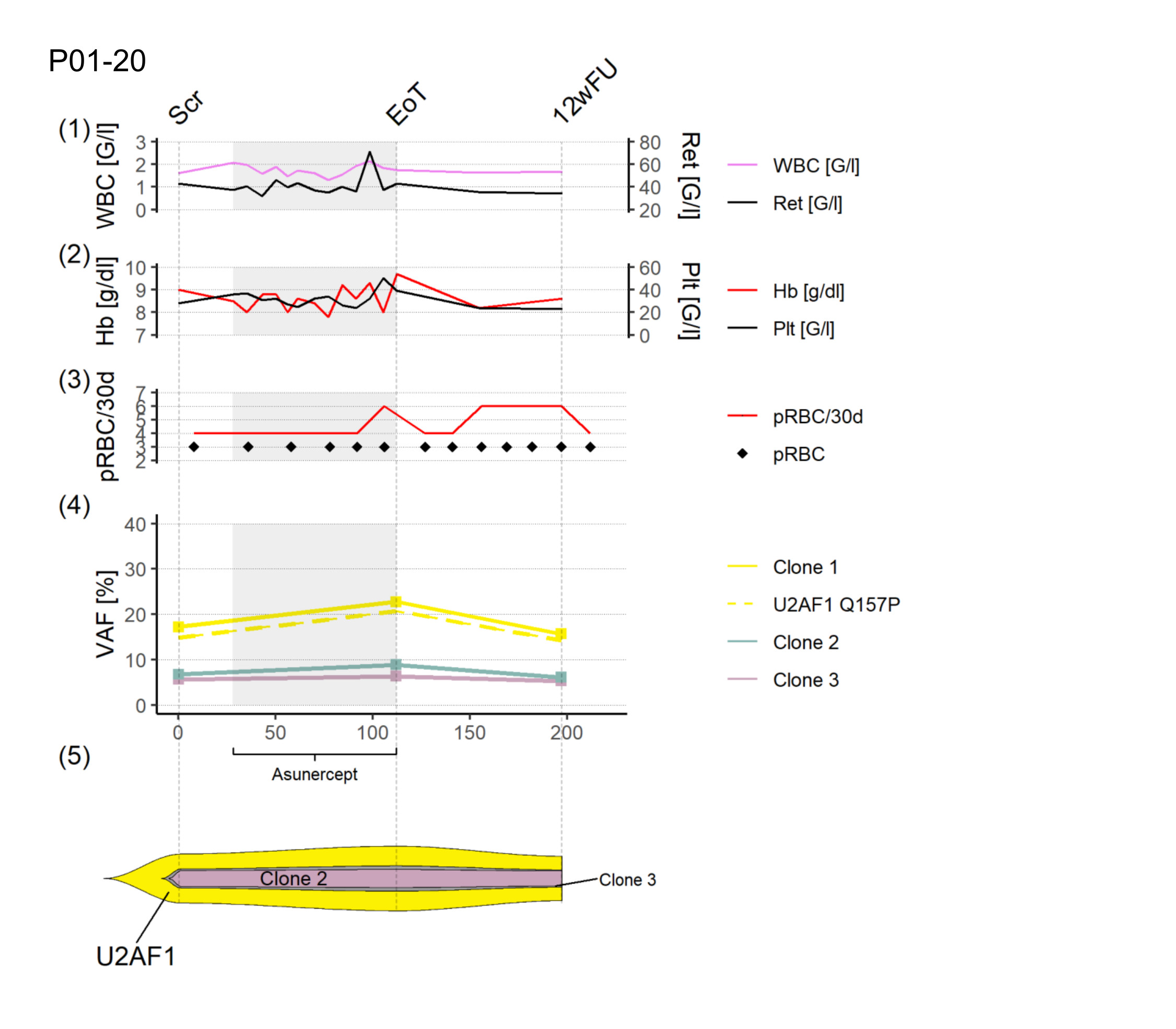

Supplement: Supplementary file 13 — (JPG 298 KB) [file 277_2024_5664_MOESM13_ESM.jpg]
